# Long-Term Genomic Surveillance and Immune Escape of SARS-CoV-2 in the Republic of Korea, with a Focus on JN.1-Derived Variants

**DOI:** 10.3390/v17091202

**Published:** 2025-08-31

**Authors:** Il-Hwan Kim, Eun Ju Lee, Jin Sun No, Ji Yeong Noh, Chae Young Lee, Sang Won O, Yong Jun Choi, Jeong-Ah Kim, Bo Min An, Jeong-Hyun Nam, Jeong-Min Kim, Jee Eun Rhee, Eun-Jin Kim

**Affiliations:** Division of Emerging Infectious Diseases, Department of Laboratory Diagnosis and Analysis, Korea Disease Control and Prevention Agency, Cheongju 28159, Republic of Korea; ilhwan98@korea.kr (I.-H.K.); znel444@korea.kr (E.J.L.); njs2564@korea.kr (J.S.N.); wldud1540@korea.kr (J.Y.N.); lcy0325@korea.kr (C.Y.L.); kay7305@korea.kr (S.W.O.); youngjun9103@korea.kr (Y.J.C.); kja88@korea.kr (J.-A.K.); bman20@korea.kr (B.M.A.); jeonghyun82@korea.kr (J.-H.N.); jmkim97@korea.kr (J.-M.K.); jerhee001@korea.kr (J.E.R.)

**Keywords:** COVID-19, SARS-CoV-2, variant, genomic epidemiology, genomic surveillance, neutralization antibodies, phylogenetic analysis

## Abstract

Since the onset of the COVID-19 pandemic, the Republic of Korea has experienced continuous waves of SARS-CoV-2 variants. The current study aimed to analyze the long-term trends of variant prevalence and associated changes in immune responses within the country. Whole-genome sequencing was performed on confirmed patient samples collected from December 2020 to May 2025, and variant distribution, genetic diversity, and neutralization were compared. As a result of analyzing a total of 157,962 gene sequences, various Omicron sub-lineages, including BA.1, BA.2, BA.5, followed by JN.1, KP.3, and NB.1.8.1, were seen to circulate sequentially over time. The nucleotide diversity of the SARS-CoV-2 genome gradually increased after the JN.1 outbreak. Of the tested variants, hamster antiserum neutralization analysis indicated that Omicron NB.1.8.1, which began to circulate in 2025, exhibited the lowest neutralization activity, with an approximately 6.6-fold decrease compared to JN.1. This suggests a potential expansion in the dominance of new variants with enhanced immune evasion. As the transmission of SARS-CoV-2 continues, new variants with novel characteristics may emerge; therefore, continuous national genomic surveillance and immunological characterization are considered crucial for early detection of emerging variants and for guiding effective public health responses.

## 1. Introduction

The coronavirus disease 2019 (COVID-19) first emerged in late 2019 and was declared a pandemic by the World Health Organization (WHO) on 11 March 2020 [1,2]. As of 2 February 2025, over 770 million cases and 7 million deaths have been reported globally [3]. Although the pandemic ended in May 2023 [4], SARS-CoV-2 variants continue to circulate, prompting ongoing global monitoring for novel variants and potential re-spread [5].

SARS-CoV-2, the causative agent of COVID-19, is a positive-sense single-stranded RNA virus that may undergo recombination, insertion, deletion, and point mutations [6,7]. These changes contribute to viral evolution, influencing transmissibility, virulence, immune escape, and vaccine effectiveness. After the Alpha variant, defined as a variant of concern (VOC) by the WHO, first appeared at the end of 2020, the Omicron variant emerged in November 2021 and spread globally with sub-lineages such as BA.2, BA.5, and XBB. Since 2024, the JN.1 lineage variants, antigenically distinct from XBB, have impacted COVID-19 incidence [8,9,10].

The Korea Disease Control and Prevention Agency (KDCA) conducts genomic surveillance using whole-genome sequencing via the Korea Respiratory Virus Integrated Surveillance System (K-RISS) [11]. This involves monitoring the emergence of new variant viruses and analyzing the genetic characteristics of SARS-CoV-2. In Korea, VOCs such as Alpha and Delta circulated in 2021, and with the introduction of Omicron, various sub-lineages, including BA.2, BA.5, BN.1, XBB, and HK.3, circulated sequentially from 2022 to 2023 [11,12].

The accumulation of mutations in diverse SARS-CoV-2 sub-lineages, particularly in the spike protein, can lead to evasion of existing immune responses, reduced vaccine and therapeutic efficacy, and recurrent outbreaks [13,14]. To assess the impact of these variants, antibody response analysis is necessary. Neutralization assays using experimentally generated animal antisera can be used to measure antigenic differences and evaluate vaccine efficacy, providing valuable information for public health responses [15,16].

Accordingly, in this study, we conducted whole-genome sequencing on confirmed patient samples to understand the overall epidemiological trends of SARS-CoV-2 in the Republic of Korea from December 2020 to May 2025. In particular, by analyzing the characteristics of variants and antibody responses after the emergence of Omicron JN.1, we aimed to enhance the understanding of SARS-CoV-2 variant characteristics and contribute to strengthening management strategies.

## 2. Materials and Methods

### 2.1. Clinical Specimens and RNA Extraction

Nasopharyngeal and oropharyngeal swabs were collected from patients with SARS-CoV-2 infection confirmed by a real-time reverse transcriptase-polymerase chain reaction (RT-PCR), as part of the Korea Respiratory Virus Integrated Surveillance System (K-RISS). Middle–high viral RNA load samples (Ct < 30 for E and N genes) were selected for genome sequencing. Total RNA was extracted from samples using a QIAamp Viral RNA mini kit (QIAGEN, Hilden, Germany) according to the manufacturer’s instructions. All specimens were handled under a biosafety cabinet according to the KDCA laboratory biosafety guidelines for COVID-19.

### 2.2. Whole-Genome Sequencing

Whole-genome sequencing was conducted on 16,623 samples. To analyze data from the initial detection of SARS-CoV-2 in the Republic of Korea from January 2020 until May 2025, 141,339 sequences from previous studies were included, resulting in the analysis of a total of 157,962 sequences [11,12].

To obtain the genomic sequences of SARS-CoV-2, libraries were prepared using the Illumina COVIDSeq RUO kits (Illumina, CA, USA) according to the manufacturer’s instructions. Sequencing was performed on the MiSeq instrument with 2 × 150 bp using a MiSeq reagent kit v2 (300 cycles) or a NextSeq instrument using NextSeq 1000/2000 P2 Reagents (200 cycles) v3 (Illumina).

Total reads were trimmed and low-quality reads were filtered using the CLC Genomics Workbench version 22.0.2 (QIAGEN Digital Insights, Aarhus, Denmark). The filtered reads were mapped to the reference sequence of SARS-CoV-2 (NC_045512.2), and consensus sequences were extracted. The SARS-CoV-2 lineages were identified using Phylogenetic Assignment of Named Global Outbreak Lineages (PANGOLIN). The sequences were submitted to the GISAID (https://www.gisaid.org/, accessed on 4 July 2025) database, and only filtered complete sequences (genomes > 29,000 nucleotides) were used in the analysis.

### 2.3. Genetic Diversity Analysis

For efficient computation and analysis, a representative dataset of 919 sequences (October 2023 to May 2025) was generated from high-quality complete sequences (>99.0% coverage) using CD-HIT at 99.5% nucleotide similarity to reduce redundancy while preserving overall genomic structure [17]. To analyze short-term evolutionary changes, representative sequences were selected for each month, with more than 1000 available sequences until April 2024, using a stricter 99.95% nucleotide similarity threshold to reflect lower genetic diversity over shorter periods. From May 2024 onward, all available sequences were analyzed due to the limited samples. Nucleotide diversity was then calculated for both the overall and month-by-month datasets using DnaSP v5 with a sliding window of 50 bp and increments of 10 bp [18].

### 2.4. Phylogenetic Analysis

For the phylogenetic analysis, Geneious Prime software (https://www.geneious.com/, accessed on 10 July 2025) was used. Complete sequences of high quality, filtered based on genome coverage (>99.0%), were aligned using MAFFT version 7 by mapping against the Wuhan-Hu-1 strain (NC_045512.2). The maximum-likelihood phylogenetic trees were generated by using FastTree version 2.1.11 under the general time-reversible plus gamma nucleotide substitution model. The phylogenetic trees were visualized using iTOL version 6 (https://itol.embl.de/, accessed on 10 July 2025).

### 2.5. Virus Isolation

Viruses were isolated from nasopharyngeal and oropharyngeal samples collected from patients with COVID-19. An amount of 100 µL of the sample was inoculated into VeroE6 cells prepared the day before, and cultured at 37 °C in 5% CO_2_ conditions in 1× Dulbecco’s modified Eagle’s medium (DMEM) supplemented with 2% fetal bovine serum and penicillin–streptomycin. Virus proliferation and isolation were confirmed using cytopathic effects and gene detection. SARS-CoV-2 virus cultivation was performed in a Biosafety Level-3 facility according to the laboratory biosafety guidelines of the KDCA.

### 2.6. Animal Study

Animal experimental protocols in this study strictly followed general animal care guidelines mandated under the Guidelines for Animal Use and Care of the KDCA. A total of 16 six-w-old female Golden Syrian hamsters (HFK Bioscience, Beijing, China) were anesthetized with ketamine-xylazine, and intranasally inoculated with 10^5^ plaque-forming units (PFU) in 100 µL of SARS-CoV-2 strains belonging to JN.1 (*n* = 9) and KP.3 (*n* = 5) lineages. Each animal was considered an experimental unit, and the number of animals per group was determined to allow for statistical analysis of antibody responses. Following virus inoculation, hamsters were monitored daily for body weights, clinical signs, and mortality for 14 d. Serum samples were collected from infected animals on day 25 post-infection and control serum was obtained from uninfected, age-matched hamsters (*n* = 2).

### 2.7. Plaque Reduction Neutralization Test

Hamster serum samples were heat-inactivated at 56 °C for 30 min and subsequently subjected to five-fold serial dilutions in culture medium, starting at a 1:5 dilution. Each diluted sample was mixed with 1000 PFU/mL of SARS-CoV-2 Omicron variants (JN.1, KP.3, XEL, KP.3.1.1, XEC, LP.8.1, and NB.1.8.1), resulting in final dilutions of 1:10 to 1:31,250. The virus–serum mixtures were incubated at 37 °C for 1 h and then inoculated onto 12-well plates pre-seeded the previous day with a monolayer of VeroE6 cells. After incubation for 2–3 days, plaques were visualized, and the highest serum dilution that resulted in ≥50% reduction in plaque formation was recorded. The neutralizing antibody titer (PRNT_50_) was defined as the reciprocal of the highest serum dilution that caused a 50% reduction in the number of plaques, compared to the virus-only control.

## 3. Results

### 3.1. Distribution of Variants in the Republic of Korea

**Figure 1:**
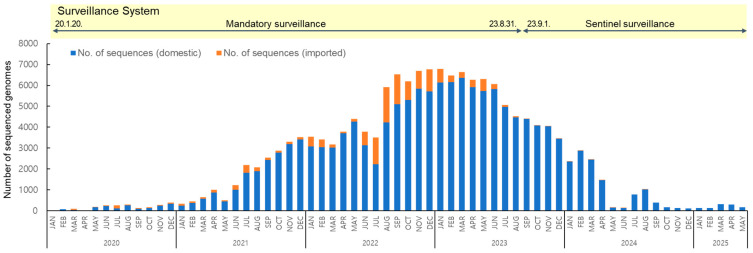
Monthly distribution of 157,962 SARS-CoV-2 sequenced genomes collected from domestic and imported cases from January 2020 to May 2025.

To analyze the long-term prevalence of SARS-CoV-2 variants from their first emergence in January 2020 to May 2025, a total of 157,962 sequences were obtained, of which 144,258 were of domestic origin and 13,704 were from overseas (Figure 1). Following the reclassification of COVID-19 under Korea’s notifiable infectious disease system from a Class 2 to Class 4 in September 2023, the surveillance system shifted from mandatory to sentinel surveillance. Class 2 infectious diseases require mandatory referral due to higher transmissibility and risk, whereas Class 4 diseases are monitored through sentinel surveillance due to their lower risk. As a result, the counting of confirmed cases was discontinued, with the total reaching 34,571,873 as of 31 August [19].

**Figure 2:**
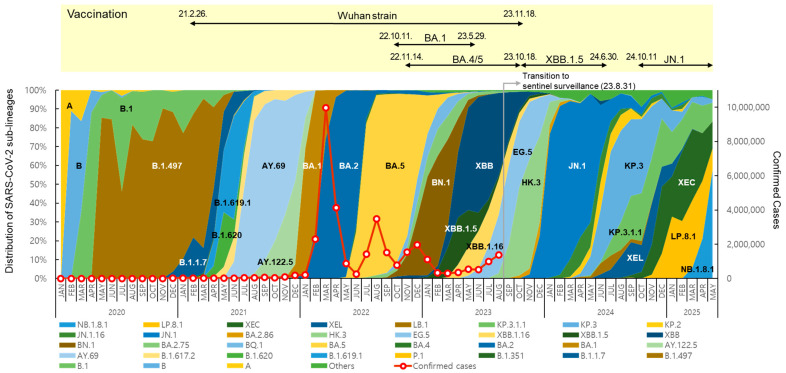
Distribution of SARS-CoV-2 sub-lineages, confirmed cases, and status of COVID-19 vaccination in the Republic of Korea. Each sub-lineage includes descendant lineages, except those individually specified elsewhere in the graph. Confirmed case counting was discontinued following the transition to sentinel surveillance (31 August 2023).

The distribution and prevalence of SARS-CoV-2 sub-lineages changed continuously over time (Figure 2). In May 2020, B.1.497, belonging to the GH clade, spread nationwide and predominated, with a prevalence of over 50% until March 2021. From July to December 2021, Delta variants, such as AY.69 and AY.122.5, were dominant (65.8% to 100%). In 2022, Omicron BA.1, BA.2, BA.5, and BN.1 circulated, and in 2023, XBB-derived variants, such as XBB.1.5, EG.5, and HK.3, circulated sequentially.

After the first domestic detection of BA.2.86 (derived from BA.2) on 31 August 2023 [12], its descendant, JN.1, increased rapidly from December, showing a prevalence of over 70% until May 2024. From July to November, JN.1-derived KP.3 and KP.3.1.1 showed a high prevalence of over 50%. From January to April 2025, JN.1-derived LP.8.1 showed a high prevalence of 30.5% to 35.8%, and the Omicron recombinant XEC also accounted for 21.4% to 37.7%. However, NB.1.8.1, derived from the Omicron recombinant XDV first detected in Korea in February 2025, tended to increase, with prevalence rates of 21.2% in April and 57.8% in May.

New vaccines were developed in response to the COVID-19 variant outbreaks, and the KDCA decided to introduce suitable vaccine strains for domestic use through the Expert Committee on Immunization Practices, which implemented vaccination programs [20]. Vaccination with Wuhan-strain-based COVID-19 vaccines first began on 26 February 2021 [21]. Subsequently, BA.1 bivalent vaccines [22] were introduced in October 2022, followed by BA.4/5 bivalent vaccines in November, and XBB.1.5 monovalent vaccines targeting XBB sub-lineages have been used since October 2023 [23,24]. Since October 2024, vaccines have been administered using JN.1 lineage monovalent vaccines [25].

### 3.2. Genetic Diversity of Variant Genomes

**Figure 3:**
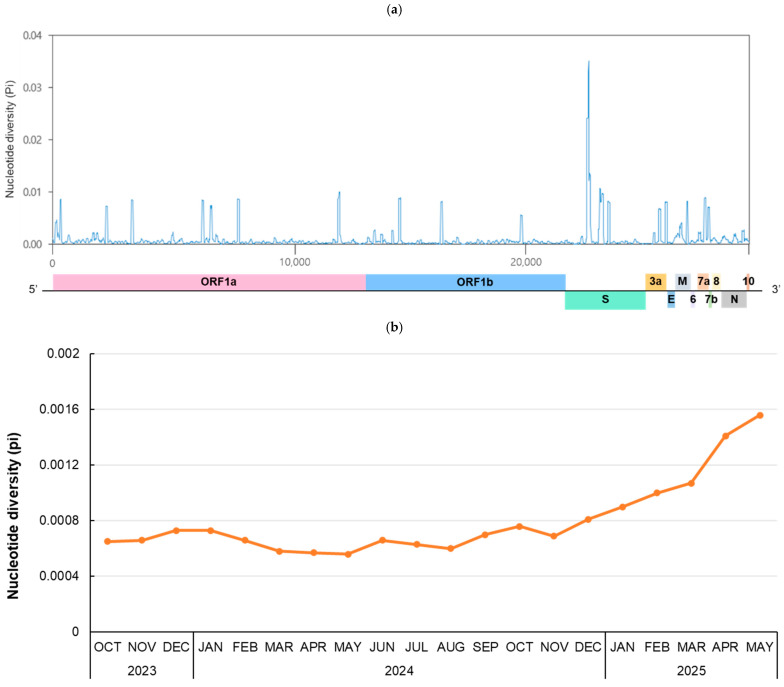
Nucleotide diversity in the SARS-CoV-2 genome. (**a**) A total of 919 genome sequences from October 2023 to May 2025 were selected using CD-HIT and analyzed to evaluate the genetic diversity of the SARS-CoV-2 genome. The nucleotide diversity (π) was calculated using a sliding window of 50 bp with 10-bp steps across the selected 1–29,379-bp region. (**b**) The mean nucleotide diversity per site is shown for each month. Sequences were selected using CD-HIT until April 2024. From May 2024 onward, all available sequences were included due to the limited samples. The number of genome sequences analyzed per month is as follows: 835 sequences (2023-Oct), 934 (2023-Nov), 951 (2023-Dec), 580 (2024-Jan), 610 (2024-Feb), 535 (2024-Mar), 1184 (2024-Apr), 161 (2024-May), 137 (2024-Jun), 783 (2024-Jul), 1034 (2024-Aug), 388 (2024-Sep), 170 (2024-Oct), 141 (2024-Nov), 113 (2023-Dec), 131 (2025-Jan), 141 (2025-Feb), 318 (2025-Mar), 297 (2025-Apr), 180 (2025-May).

To investigate genetic diversity during the 20-month period from October 2023, when JN.1 began to circulate, to May 2025, RNA sequence polymorphism was analyzed (Figure 3). For the whole genome (29,379 bp), the number of polymorphic segregating sites and total number of mutations were 1883 and 1964, respectively. There were 819 haplotypes, and the haplotype diversity (Hd) was 0.9994. The number of nucleotide polymorphisms per site (θ_w_) was 0.01420, and the nucleotide diversity per site (π) was 0.00098. The highest peak π value, 0.03515, was observed at positions 22,066–22,157 bp, which is part of the N-terminal domain of the spike gene (Figure 3a). To investigate the monthly pattern of RNA sequence polymorphism, the monthly nucleotide diversity of the whole genome from October 2023 to May 2025 was calculated (Figure 3b). The mean of π value for each month increased continuously after December 2024, peaking at 0.00156 in May 2025.

### 3.3. Phylogenetic Analysis of Variants

To genetically characterize Omicron JN.1 sub-lineages that occurred in the Republic of Korea, lineage analysis was conducted on whole-genome sequencing data from October 2023 to May 2025. A total of 725 complete sequences were selected, and phylogenetic analysis was then conducted (Supplementary Figure S1). Phylogenetic analysis results confirmed that JN.1, KP.3, KP.3.1.1, XEL, XEC, LP.8.1, and NB.1.8.1 each formed distinct clusters. KP.3, KP.3.1.1, LP.8.1, XEL, and XEC were confirmed to belong to the JN.1 sub-lineage and form a monophyletic group.

### 3.4. Plaque Reduction Neutralization Test

**Figure 4:**
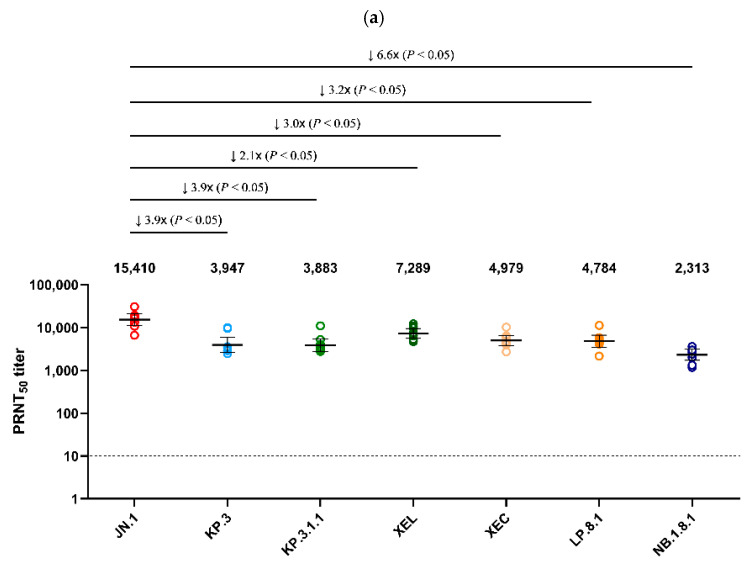

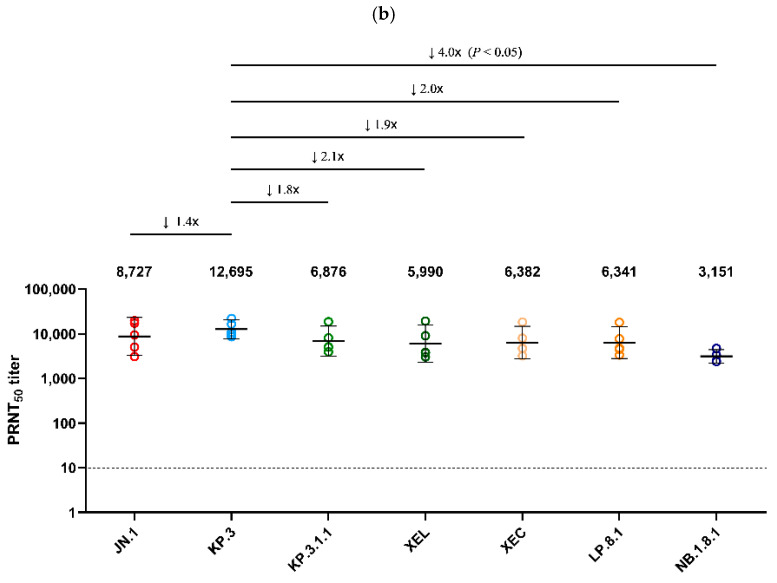
Comparison of neutralizing antibody titers against Omicron sub-lineages in hamster antisera. (**a**) PRNT_50_ results for JN.1-infected hamster antisera (*n* = 9) and (**b**) for KP.3-infected hamster antisera (*n* = 5) are presented. Neutralizing antibodies were evaluated against JN.1, KP.3, KP.3.1.1, XEL, XEC, LP.8.1, and NB.1.8.1 isolates. Each individual dot represents the geometric mean titer (GMT) obtained from two independent experiments (two replicates each). Lines and error bars indicate the group GMT and 95% confidence interval, respectively. Numbers at the top of each graph denote the GMT of neutralizing antibodies, with arrows indicating fold reduction relative to JN.1 and KP.3. The PRNT_50_ baseline is shown as a dashed line (value = 10). *p*-values were calculated using the non-parametric Mann–Whitney U test with Prism version 9 (GraphPad Software, San Diego, CA, USA). Abbreviations: GMT, geometric mean titer; PRNT_50_, 50% plaque reduction neutralization titer.

Using serum collected on day 25 from Golden Syrian hamsters infected with JN.1 and KP.3 viruses, a PRNT_50_ was performed against the seven major Omicron sub-lineages detected in Korea after 2024 (JN.1, KP.3, XEL, KP.3.1.1, XEC, LP.8.1, NB.1.8.1) (Figure 4). JN.1 infected serum showed the highest neutralization activity (GMT 15,410) against the autologous JN.1 virus (Figure 4a). Neutralization against NB.1.8.1 (GMT 2313) was the lowest, with an approximately 6.6-fold decrease compared to JN.1 (*p* < 0.05). XEL, KP.3.1.1, XEC, and LP.8.1 decreased 2.1-, 3.9-, 3.0-, and 3.2-fold, respectively, compared to JN.1 (*p* < 0.05). In the analysis of antiserum from hamsters infected with KP.3, which circulated in Korea after JN.1, the highest neutralization activity (GMT 12,695) was also observed against the autologous KP.3 virus. Of these, neutralization against NB.1.8.1 (GMT 3151) was the lowest, with a 4.0-fold decrease compared to KP.3 (*p* < 0.05) (Figure 4b).

## 4. Discussion

In this study, a comprehensive analysis of the genetic epidemiology of SARS-CoV-2 circulating in the Republic of Korea was conducted through genomic surveillance performed by the KDCA from January 2020 to May 2025. Special attention was given to analyzing the genetic characteristics and antibody responses of sub-lineages identified after the emergence of Omicron JN.1.

In the Republic of Korea, after the first COVID-19 case was confirmed on 20 January 2020 [26], national social distancing measures were implemented from 29 February. Quarantine measures, including mandatory 14-day isolation for all arrivals, were implemented from April 2020 [27]. As a result, B.1.497 and Delta AY.69, AY.122.5, which were distinct from overseas trends, maintained dominance for a prolonged period from 2020 to the end of 2021 [11]. However, with the introduction of the highly transmissible Omicron variant in November 2021, confirmed cases surged until March 2022, and the patterns of COVID-19 incidence and variant prevalence in Korea and abroad became similar [28]. Subsequently, the number of confirmed cases decreased sharply; with high vaccination rates and the availability of countermeasures such as antivirals, quarantine measures were eased, including the lifting of social distancing in April 2022. Until 2023, sub-lineages such as BA.1, BA.2, BA.5, BN.1, XBB, EG5, and HK.3 circulated sequentially in Korea [11,12].

BA.2.86, confirmed to have entered Korea in August 2023, had 36 more mutations in its spike protein than the circulating XBB.1.5, showing antigenically distinct characteristics [10]. JN.1, derived from BA.2.86, possessed an additional L455S mutation in its spike protein, and exhibited high transmissibility and immune evasion characteristics. Consequently, the JN.1 sub-lineage rapidly became dominant both domestically and internationally, leading the WHO to recommend the use of JN.1 vaccines [29].

In Korea, JN.1, KP.3, KP.3.1.1, XEL, XEC, LP.8.1, and NB.1.8.1 circulated sequentially after 2024. The nucleotide diversity (π = 0.00098) analyzed in Korean variants circulating after JN.1 was lower than that during the domestic XBB outbreak (π = 0.00155) [12], but still higher than the previously reported global nucleotide diversity (π = 0.00044) [30]. Furthermore, the mean of π for each month increased continuously after December 2024, peaking at 0.00156 in May 2025. The nucleotide diversity of a virus reflects the accumulation of genetic changes over time, and SARS-CoV-2 frequently undergoes mutations in the spike protein gene, generating new antigenic variants that can enhance the virus’s ability to evade antibodies and other immune defenses. This observed increase in nucleotide diversity was presumed to be related to the increasing prevalence of variants such as XEC, LP.8.1, and NB.1.8.1, and the increase in variants showing enhanced immune evasion [31]. After May 2024, the number of available sequences was limited; thus, all sequences were included without CD-HIT clustering to reduce potential bias. While caution is needed in the interpretation of our results, the impact on the observed increase in diversity is unlikely to be substantial, as π value mainly reflects underlying genetic differences rather than sample size [32].

Hamster antisera production can be approved by animal ethics committee less strictly, whereas human sera are more difficult to obtain due to ethical issues. Furthermore, hamster antisera can be produced against single variants, which is not possible for human sera. Although the hamster immune system is not identical to that of humans, it shows a similar neutralization trend to human sera, making it useful for predicting human neutralization [16,33]. Therefore, cross-neutralization against major Omicron sub-lineages detected in Korea after 2024 was evaluated using antisera from hamsters infected with JN.1 and KP.3. The results showed a significant reduction (*p* < 0.05) in neutralization activity of JN.1-infected antisera against other viruses, excluding JN.1. A similar decrease in neutralizing antibody titers was also observed for KP.3-infected antisera. Among all viruses tested, NB.1.8.1 exhibited the lowest neutralization activity against both JN.1- and KP.3-infected antisera, showing 6.6-fold and 4.0-fold reductions, respectively. The observed reduction in neutralization activity is consistent with the increased prevalence of NB.1.8.1, suggesting that its high immune evasion contributed to its spread. Similar results for the reduction in NB.1.8.1 neutralization activity have also been reported in a recent study using pseudo-viruses [34,35].

NB.1.8.1, a descendant of Omicron recombinant XDV derived from XBB and JN.1 [35], is currently being monitored as a “Variant Under Monitoring” by the WHO, and its increased incidence in Korea is also considered to be related to the increasing pattern in Asian regions, such as China and Hong Kong (Supplementary Figure S2) [34,35,36]. Amino acid mutations in NB.1.8.1′s spike protein, such as V445H, A435S, and T478I, have been reported to be associated with increased transmissibility and immune evasion. However, previous antigenic map analyses have shown that NB.1.8.1 forms the same cluster as the JN.1 sub-lineage, suggesting that the currently used JN.1 vaccine is expected to remain effective [34,37]. The WHO has also reported that no association with increased public health risk or severity has been confirmed so far [36]. Although neutralization titers may be maintained above protective levels, NB.1.8.1 has functional advantages, such as accumulated antibody-escaping mutations and increased ACE2 binding affinity, necessitating continuous surveillance. Considering that the United States Food and Drug Administration and WHO recommended the adoption of LP.8.1 lineage-based vaccines in May 2025, and LP.8.1 and NB.1.8.1 are currently circulating in Korea, it is necessary to review the utility of introducing new vaccines [38,39].

This study, like other national surveillance efforts, may have a sampling bias. Following the transition from mandatory to sentinel surveillance and the subsequent decrease in the number of reported positive cases, there is a possibility of underestimating the proportion of circulating viral lineages. This may also make it difficult to capture the subtle epidemiological changes between viral lineages. Despite these limitations, the KDCA continues to prioritize performing whole-genome analysis to obtain reliable data, even as the global number of sequenced cases declines.

To complement these efforts, the KDCA is conducting additional surveillance through SARS-CoV-2 variant analysis in airport/port and local wastewater. In parallel, it is maintaining international cooperation for next-pandemic preparedness by continuously sharing sequence data in global databases such as GISAID [40].

The KDCA has conducted national genomic surveillance for COVID-19, detecting and monitoring circulating variants in the Republic of Korea. Through immunological analysis, potential impacts of immune evasion by variants have been analyzed, providing various scientific information for public health responses. Although the COVID-19 pandemic has ended, JN.1, which is distinct from previously circulating variants, has emerged and spread, and various JN.1-derived variants and recombinants are still circulating worldwide, indicating the possibility that new variants could again threaten public health. Therefore, continuous genomic surveillance, monitoring, and characterization are crucial for early recognition of novel variants and preparedness for their spread, which are essential in public health responses.

## Data Availability

The SARS-CoV-2 whole-genome sequences were shared through the GISAID’s EpiCoV (http://www.gisaid.org). The dataset of SARS-CoV-2 complete genome sequences is available as Supplementary File S1.

## Supplementary Materials

The following supporting information can be downloaded at: https://www.mdpi.com/article/10.3390/v17091202/s1: Figure S1: Phylogenetic tree of the 725 SARS-CoV-2 genomes from confirmed cases circulating from October 2023 to May 2025. The tree was inferred with the approximate maximum-likelihood method as implemented by FastTree and was populated using iTOL. Pangolin lineages are indicated by the background color of the tips. Outer strips indicate the case and wave. Figure S2. Distribution of SARS-CoV-2 sub-lineages by continents. The proportions of SARS-CoV-2 sub-lineages for each continent were calculated using all available sequences from GISAID from 1 December 2024 to 31 May 2025 (as of 11 July 2025). GISAID, global initiative on sharing all influenza data; File S1: dataset of SARS-CoV-2 complete genome sequences submitted to the GISAID database.

## Abbreviations

COVID-19	coronavirus disease 2019
DMEM	Dulbecco’s modified Eagle’s medium
GMT	geometric mean titer
KDCA	Korean Disease Control and Prevention Agency
K-RISS	Korean Respiratory Virus Integrated Surveillance System
PFU	plaque-forming units
PRNT50	50% plaque reduction neutralization titer
RT-PCR	real-time reverse transcriptase-polymerase chain reaction
SARS-CoV-2	severe acute respiratory syndrome coronavirus 2
VOC	variant of concern
WHO	World Health Organization
