# Supplementary material for: Long-Term Genomic Surveillance and Immune Escape of SARS-CoV-2 in the Republic of Korea, with a Focus on JN.1-Derived Variants
Source: Viruses. 2025 Aug 31;17(9):1202. doi: 10.3390/v17091202 (PMC12474168; doi:10.3390/v17091202)
Supplement: Supplementary file 1 [file viruses-17-01202-s001.zip › File S1.pdf]

SUPPLEMENTAL TABLE

Data Availability

GISAID Identifier:	EPI_SET_250704wm
DOI:	https://doi.org/10.55876/gis8.250704wm

All genome sequences and associated metadata in this dataset are published in GISAID's EpiCoV database. To view the contributors of each individual sequence with details such as accession number, Virus name, Collection date, Originating Lab and Submitting Lab and the list of Authors, visit EPI_SET_250704wm

Data Snapshot

EPI_SET_250704wm is composed of 157,962 individual genome sequences.
The collection dates range from 2020-01-25 to 2025-05-31;
Data were collected in 1 countries and territories.
