# Supplementary figures and images for: Long-Term Genomic Surveillance and Immune Escape of SARS-CoV-2 in the Republic of Korea, with a Focus on JN.1-Derived Variants
Source: Viruses. 2025 Aug 31;17(9):1202. doi: 10.3390/v17091202 (PMC12474168; doi:10.3390/v17091202)

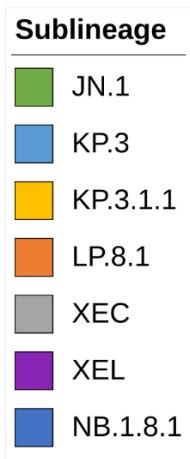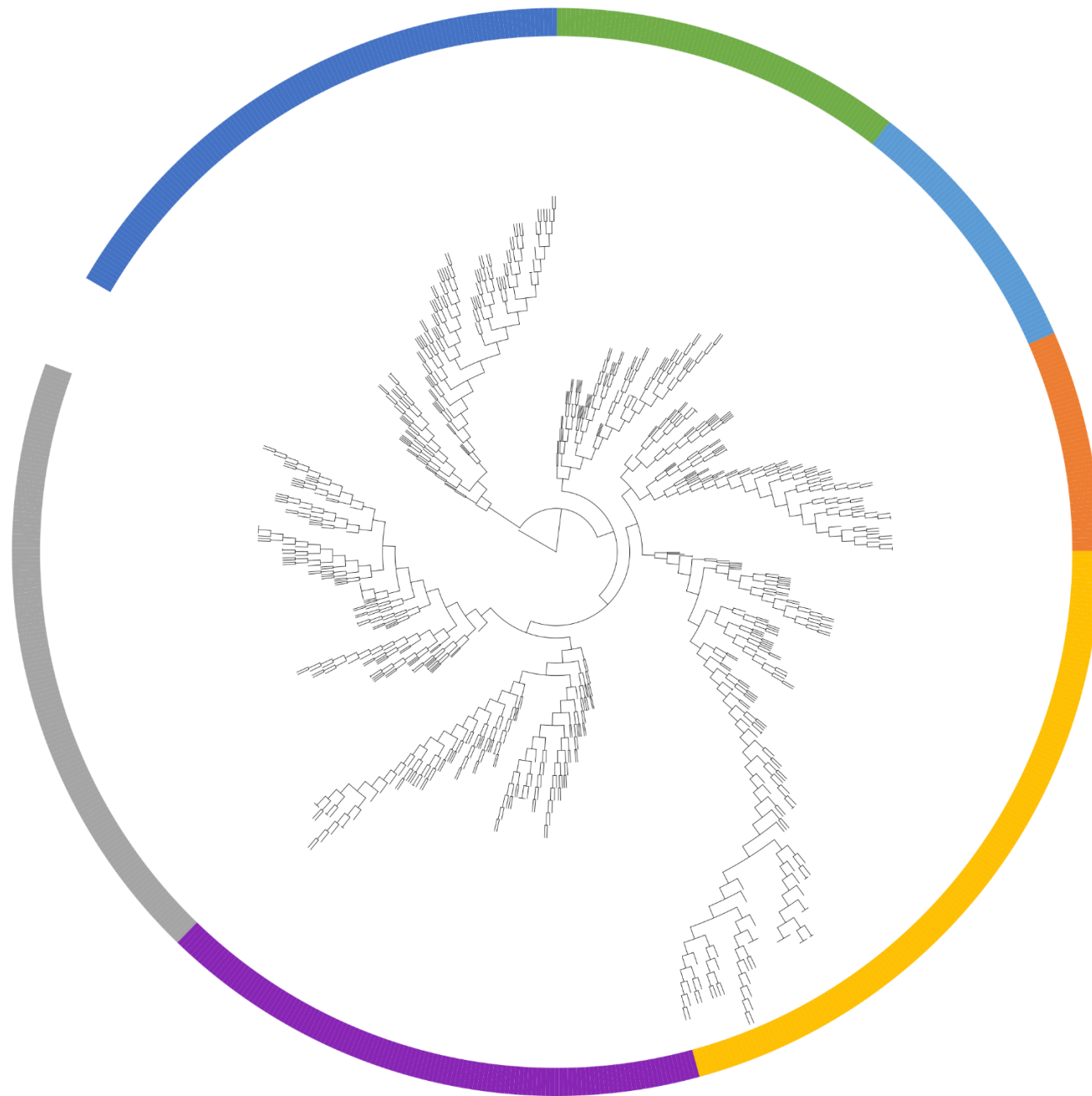

Supplement: Supplementary file 1 [file viruses-17-01202-s001.zip › Figure S1.pdf]

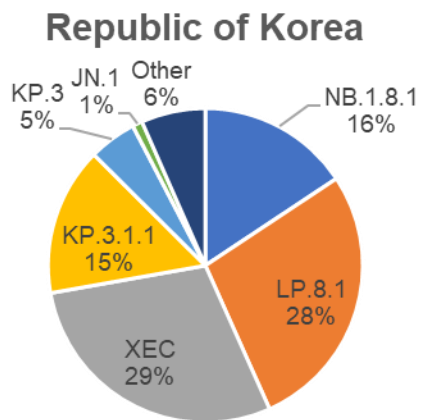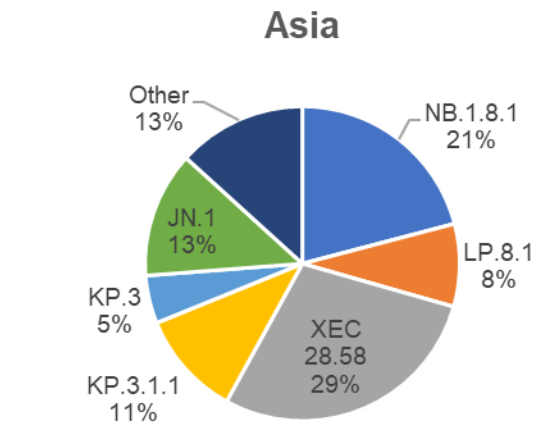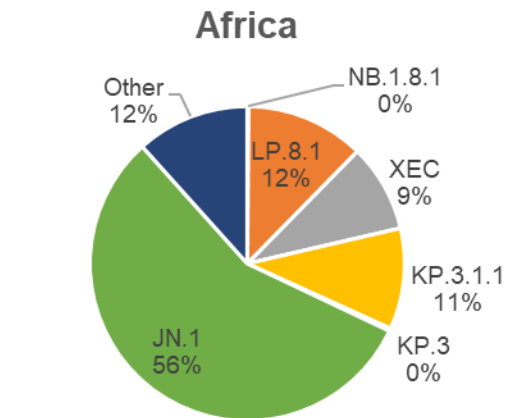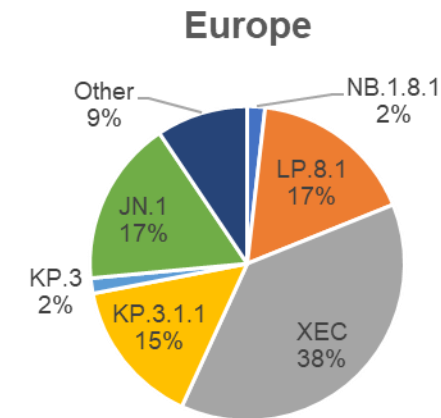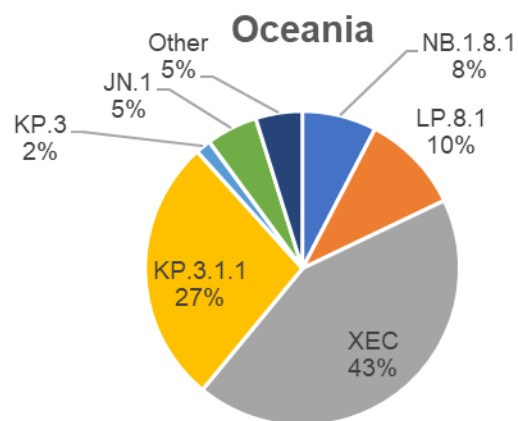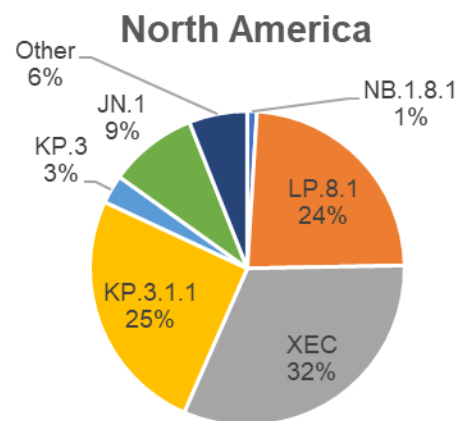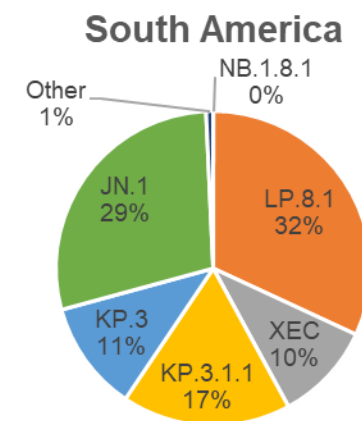

■ NB.1.8.1 ■ LP.8.1 ■ XEC ■ KP.3.1.1 ■ KP.3 ■ JN.1 ■ Other

Supplement: Supplementary file 1 [file viruses-17-01202-s001.zip › Figure S2.pdf]
